# PD31 The Significant Societal And Environmental Impact Of Chronic Kidney Disease Over The Next Decade In Brazil

**DOI:** 10.1017/S0266462324002940

**Published:** 2025-01-07

**Authors:** Talita Gobbi, Ricardo Moreira, Cinthia Montenegro, Ana Toledo-Chávarri, Naveen Rao, Stacey Priest, Stephen Brown, Hannah Guiang, Ming-Hui Zhao, Steven Chadban

## Abstract

**Introduction:**

Chronic kidney disease (CKD) is a progressively worsening condition that is often overlooked in its early stages. In Brazil, factors such as population aging and rising comorbidities are expected to shift CKD prevalence toward more advanced stages, leading to greater socioeconomic and environmental impacts. The significant burden of renal replacement therapy (RRT) suggests the need to prioritize preventive and early detection strategies.

**Methods:**

We developed a patient-level simulation model to estimate the impact of CKD in Brazil over 10 years (from 2023 to 2032) on clinical, patient, health system, environmental, productivity, and societal outcomes. Validation was conducted against Brazilian demographic data and cross-validated with the Inside CKD model. We estimated productivity losses by multiplying CKD-related workdays missed by daily costs for patients and caregivers.

**Results:**

The number of Brazilians with CKD was projected to increase by 7.2 percent (approximately 27.7 million) over the next 10 years, mainly among patients with late-stage disease, while the number of patients undergoing dialysis was projected to increase by 170.8 percent (approximately 233,000) over the same period. CKD was projected to result in BRL198 billion (USD 38 billion) of lost income. From an environmental standpoint, freshwater consumption, fossil fuel depletion, and carbon dioxide emissions due to patients with CKD were projected to increase by 40 percent by 2032. RRT was projected to require the equivalent annual water usage of approximately 370,000 households and the annual power of approximately 11 million lightbulbs and will produce annual carbon dioxide emissions equivalent to approximately 1.5 million cars.

**Conclusions:**

While the overall number of patients with CKD will increase by 7 percent (from 25.8 million in 2022 to 27.7 million in 2032), the distribution toward later stages of CKD will cause significant impacts in terms of the healthcare system (resource use and costs), patients and caregivers, society, and the environment.

